# Neonatal persistent pulmonary hypertension related to a novel *TBX4* mutation: case report and review of the literature

**DOI:** 10.1186/s13052-024-01575-3

**Published:** 2024-03-05

**Authors:** Chiara Maddaloni, Sara Ronci, Domenico Umberto De Rose, Iliana Bersani, Francesca Campi, Matteo Di Nardo, Francesca Stoppa, Rachele Adorisio, Antonio Amodeo, Alessandra Toscano, Maria Cristina Digilio, Antonio Novelli, Giovanni Chello, Annabella Braguglia, Andrea Dotta, Flaminia Calzolari

**Affiliations:** 1 Neonatal Intensive Care Unit, “Bambino Gesù” Children’s Hospital IRCCS, Rome, Italy; 2 Paediatric Intensive Care Unit and ECMO Team, “Bambino Gesù” Children’s Hospital IRCCS, Rome, Italy; 3 Heart Failure, Transplant and Mechanical Cardiocirculatory Support Unit, Department of Paediatric Cardiology and Cardiac Surgery, Heart Lung Transplantation, ERN GUARD HEART, “Bambino Gesù” Children’s Hospital IRCCS, Rome, Italy; 4 Perinatal Cardiology Unit, “Bambino Gesù” Children’s Hospital IRCCS, Rome, Italy; 5 Medical Genetics Unit, “Bambino Gesù” Children’s Hospital IRCCS, Rome, Italy; 6 Translational Cytogenomics Unit, Multimodal Medicine Research Area, “Bambino Gesù” Children’s Hospital IRCCS, Rome, Italy; 7 Neonatal Intensive Care Unit, Monaldi Hospital, Naples, Italy

**Keywords:** Neonatal persistent pulmonary hypertension, T-Box factor 4, Neonatal lung disease, ECMO, Next-generation sequencing

## Abstract

*TBX4* gene, located on human chromosome 17q23.2, encodes for T-Box Transcription Factor 4, a transcription factor that belongs to the T-box gene family and it is involved in the regulation of some embryonic developmental processes, with a significant impact on respiratory and skeletal illnesses. Herein, we present the case of a female neonate with persistent pulmonary hypertension (PH) who underwent extracorporeal membrane oxygenation (ECMO) on the first day of life and then resulted to have a novel variant of the *TBX4* gene identified by Next-Generation Sequencing. We review the available literature about the association between PH with neonatal onset or emerging during the first months of life and mutations of the TBX4 gene, and compare our case to previously reported cases. Of 24 cases described from 2010 to 2023 sixteen (66.7%) presented with PH soon after birth. Skeletal abnormalities have been described in 5 cases (20%). Eleven cases (46%) were due to *de novo* mutations. Three patients (12%) required ECMO. Identification of this variant in affected individuals has implications for perinatal and postnatal management and genetic counselling. We suggest including *TBX4* in genetic studies of neonates with pulmonary hypertension, even in the absence of skeletal abnormalities.

## Introduction

*TBX4* (T-box 4) gene, which codes for a member of the evolutionarily conserved family of T-box-containing transcription factors, has a significant impact on respiratory illnesses [1, 2]. The link of *TBX4* variations with congenital diseases affecting the respiratory and skeletal systems highlights its relevance during development. Nevertheless, the precise function of *TBX4* in human development is still not fully understood [3].

T-Box genes are DNA-binding transcription factors essential for embryogenesis [4]. *TBX4* gene (OMIM *601,719) is located on chromosome 17q23.2 and was firstly identified in 1996. In 2004, *TBX4* was recognized as responsible for Small Patella Syndrome (SPS), also known as ischial-coxo-podo-patellar syndrome (ICPPS, MIM number 147,891), a spectrum of limb and skeletal abnormalities that results in developmental abnormalities of the foot and pelvis as well as patellar aplasia or hypoplasia [5].

In 2013, Kerstjens-Frederikse et al. [6] discovered a link between pulmonary hypertension (PH) in neonates and children with mutations in the *TBX4* gene.

We present the case of a neonate with persistent PH supported with extracorporeal membrane oxygenation (ECMO) in the first day of life. Genetic analysis identified a likely pathogenetic novel heterozygous variant of the *TBX4* gene (c.1160delC). The aim of this manuscript is to describe the clinical scenario and follow-up of this patient and compare this case to previously reported cases of neonatal PH associated to *TBX4* mutations.

## Case report

### Clinical report during hospitalization

After a normal pregnancy, a 33-year-old gravida delivered at 39 weeks of gestational age a female neonate via elective caesarean section due to podalic presentation. She displayed good extrauterine life adaption at birth (Apgar scores of 8 at 1 and 9 at 5 min). At 15 min of life she experienced severe bradycardia, which led to cardio-respiratory arrest. Advanced cardiopulmonary resuscitation maneuvers involving cardiac massage and adrenaline were carried out and alprostadil infusion was started in the suspicion of duct-dependent congenital heart disease. Echocardiography revealed indirect signs of severe pulmonary hypertension (PH) with exclusive right-to-left flow via the ductus arteriosus, in the absence of major congenital heart disease. Inhaled nitric oxide (iNO) was started, surfactant was administered and inotropic support was initiated due to developing systemic hypotension.

At about 12 h of life, due to the persistence of severe PH refractory to maximal medical therapy, veno-arterial ECMO was started.

The patient received inotropic support with adrenaline, pulmonary vasodilators (iNO and sildenafil), and milrinone for concurrent biventricular dysfunction while being supported on ECMO. On the second and fifth days of life, two further doses of surfactant were also administered to improve lung compliance and speed up recovery. She was decannulated at 10 days of life under iNO and inotropic support which were gradually reduced overtime with the improvement of the cardiac function.

At 29 days of life the patient was extubated and supported with non-invasive ventilation (non-invasive positive pressure ventilation and after few days nasal continuous positive pressure). Therapy with sildenafil was continued and, at 34 days of life, given the persistence of episodes of desaturation in non-invasive ventilation and echocardiographic signs of moderate pulmonary hypertension, bosentan therapy was added. At 84 days of life, she was breathing on room air with adequate saturation.

Due to the atypical and persistent PH, extensive work up was undertaken. Infectious disease and metabolic extensive screening resulted negative. Chest computed tomography (CT) performed at 27 days of life showed a diffuse increase in lung density in the absence of areas of consolidation/atelectasis, it also excluded any tracheal abnormalities. Cardiac catheterization at 65 days of life demonstrated persistence of precapillary pulmonary hypertension (right ventricular end-diastolic pressure 9 mmHg; pulmonary artery pressure 64/30(46) mmHg; pulmonary arterial wedge pressure 10 mmHg; pressure in the ascending and descending aorta 70/34(55)mmHg), which was partly caused by the presence of a large patent ductus arteriosus. A test of PDA closure using a balloon was attempted while the patient was catheterized, yet it significantly affected the saturation patterns. The pulmonary vascular resistance calculation was not possible at first due to patent ductus arteriosus and after the attempt to close due to the impairment of saturation trends.

### Genetic analysis

Genomic DNA was obtained from the proband. Array comparative genomic hybridization (array-CGH) analysis revealed a normal karyotype. Using the Trusight ONE kit for clinical exome from Illumina, Next-Generation Sequencing (NGS) was carried out with a focus on the genes causing neonatal respiratory failure as surfactant deficiency, interstitial, and related diseases: *FOXF1* (NM_001451), *SFTPA1* (NM_005411), *SFTPA2* (NM_00109866), *ACVRL1* (NM_000020), *BMPR2* (NM_001204), *CBLN2* (NM_182511), *CRHR1* (NM_001145146), *ENG* (NM_000118), *SMAD9 (*NM_005905), *TBX4* (NM_018488), *SMAD1* (NM_005900), *PPARG* (NM_015869), *MEOX2* (NM_005924), *CSF2RB* (NM_000395), *CSF2RA*(NM_172245.4) *NKX2-1* (NM_001079668.2), *SMAD5* (NM_005903), *ABCA3* (NM_001089), *SFTPC* (NM_003018), *SFTPB* (NM_000542), *SFTPD* (NM_003019), *SLC7A7* (NM_001126106), *MARS1* (NM_004990.4), *GATA2* (NM_032638.5), *OAS1* (NM_016816.4), *KCNK3* (NM_002246), *CAV1* (NM_001753), *EIF2AK4* (NM_001013703), *SLC34A2* (NM_006424.2), *MUC5B* (NM_002458.3).

The Illumina NextSeq 550 platform with a sequencing depth of 100X was used to accomplish DNA capture, enrichment, and paired-end sequencing with a read length of 149 bp. The variations were annotated using the data processing tool Illumina VariantStudio 3.0. The NGS study revealed the novel heterozygous frameshift TBX4 mutation (NM_018488 (TBX4): c.[1160delC];[=] p.[(Arg389fsTer27)];[=] which at the protein level determines the introduction of the premature stop codon p.Arg389fsTer27 (rs1569046598). To confirm the mutations discovered by NGS in the proband, conventional Sanger sequencing was carried out using an ABI 3130xl capillary sequencer (Applied Biosystem). The frameshift variant was inherited from the father, with autosomal dominant inheritance.

This new variant c.1160delC has never been described in the literature or reported in public reference databases (i.e. Genome Aggregation Database: accessible on https://gnomad.broadinstitute.org/; dbSNP: accessible on https://www.ncbi.nlm.nih.gov/snp/); it can be classified according to the ACMG guidelines as a probably pathogenic variant (class 4).

We looked into the family history after the genetic data became available: the father was perfectly asymptomatic and no other family members displayed any skeletal, cardiac or pulmonary problems.

### Follow up

The infant is currently 10 months old, she is on therapy with oral sildenafil, bosentan, furosemide and spironolactone maintaining normal oxygen saturation level on room air. The patient is able to feed entirely orally with an adequate weight gain.

Last cardiologic evaluation, at 9 months of age, showed good biventricular function, bidirectional shunt on the fossa ovale, small patent ductus arteriosus with exclusive left-to-right shunt with continuous velocity and doppler pattern (maximal aorto-pulmonary Gmax 25 mmHg).

Cardiac catheterization at 9 months of life demonstrated an improvement of precapillary pulmonary hypertension: right ventricular end-diastolic pressure 6 mmHg; pulmonary artery pressure 40/18(24) mmHg; pulmonary arterial wedge pressure 9 mmHg; pressure in the ascending and descending aorta 70/40(50)mmHg, cardiac index 2.33 l/min/m^2^, pulmonary vascular resistance index 6.43 WU*m^2^; systemic vascular resistance index 18.45 WU*m^2^.

Skeletal X Ray examination excluded any signs of bone disease according to the age of the patient.

## Review of the literature

In Table 1 we summarized previously described cases of pulmonary hypertension with neonatal onset or emerged during firsts months of life associated to *TBX4* mutations. Overall we found twenty-four cases described from 2010 to 2023 [7]– [10, 1, 11]– [13, 14]– [17–20], 16 of them presented with PH soon after birth [7]– [10, 12, 14]– [20]. Nine/24 infants (37.0%) were males. Skeletal abnormalities have been described in 5 cases (20%), affecting toes, limb and pelvis; facial dysmorphic features were evident in 4 neonates (16,5%) and in 4 cases (16,5%) a neurodevelopmental delay was described.

**Table 1:** Cases of TBX4 mutation with neonatal onset pulmonary hypertension or pulmonary hypertension emerging during firsts months of life. CT: computed tomography, ECMO: extracorporeal membrane oxygenation, iNO: inhaled nitric oxide, NA: not applicable, O2: oxygen, PH: pulmonary hypertension

	First author, year	Sex	Onset	Gene mutation	Position in protein and amino acids	Inheritance	Others clinical features (0 no;1 toes-pelvis abnormalities, 2 dysmorphic features; 3 neurodevelopmental delay)	Diagnostic approach	Response to medical therapy (+,+/-,-)	Type of drugs used for pulmonary hypertension and cardiovascular dysfunction	Surgical treatment	Age and outcome at follow up
1	Nimmakayalu,2010 [7]	F	Birth	microdeletion of17q22q23.2 encompass- ing and TBX4	NA	De novo	2	Echocardiography	NA		tracheostomy, percutaneous gastrostomy	3 years: improvement of the pulmonary hypertension
2	Szafranski, 2016 [8]	F	Birth	p.E86Q (c.256G > C)	NA	De novo	NA	Echocardiography	NA		none	Neonatal death (first day of life)
3	Suhrie, 2019 [9]	F	Birth	c.524_527del	p.Asn175Thrfs*52	De novo	0	Echocardiography, autopsy	-	iNO, milrinone,inhaled epoprostenol	ECMO	19 days: death
4	Suhrie, 2019 [9]	M	Birth	Del cr 17 (17q23.1-17q23.2)	NA	De novo	0	Echocardiography, CT, lung biopsy, cardiac catheterization	-	O2	tracheostomy, percutaneous gastrostomy	15 months: chronic respiratory failure
5	German, 2019 [10]	M	Birth	Del cr 17 (17q23.2 to 17q23.2)	NA	De novo	1, 2	Echocardiography, lung biopsy	-	O2, iNO	ECMO	10 days: death
6	Liu, 2019 [19]	F	Birth	c.1633G > T	p.G545X	AD	NA	Echocardiography	NA		NA	NA
7	Chi, 2019 [20]	NA	Birth	Del TBX gene	NA	NA	NA	Echocardiography, lung biopsy	+/-	iNO, sildenafil, milrinone, epoprostenol	ECMO	1 month: improvement of PH
8	Galambos, 2019 [11]	M	3 m	Del 17q23.1q23.2	NA	De novo	0	Echocardiography, biopsy	+/-	O2, phosphodiesterase 5 inhibitor	none	5 months death
9	Galambos, 2019 [11]	F	2 m	Del 17q23.1q23.2	NA	NA	1,3	Echocardiography	+	phosphodiesterase 5 inhibitor, endothelin receptor antagonist	none	10 years: stable mild PH
10	Galambos, 2019 [11]	F	2 m	Del 17q23.1q23.2	NA	NA	1,3	Echocardiography	+	O2, phosphodiesterase 5 inhibitor,	none	15 years: stable PH
11	Galambos, 2019 [11]	F	1 m	c.251_delG p.	(Gly84Alafs*4)	De Novo	3	Echocardiography	+/-	O2, iNO, phosphodiesterase 5 inhibitor,	none	8 months: death, PH crisis during surgery (Meckel’s diverticulectomy)
12	Galambos, 2019 [11]	F	1 m	c.1018 C > T p.(Arg340* )	(Arg340*)	NA	0	Echocardiography, biopsy	+/-	O2, phosphodiesterase 5 inhibitor,	none	4 years: severe lung disease, mild PH
13	Galambos, 2019 [11]	M	1 m	c.1018 C > T p.(Arg340*)	(Arg340*)	NA	0	Echocardiography	+	O2, phosphodiesterase 5 inhibitor, endothelin receptor antagonist	none	3 months no PH, short follow-up
14	Karolak, 2019 [12]	M	Birth	c.1198G > A (p.Glu400Lys)	(p.Glu400Lys)	AD	2	Echocardiography, chest CT, lung biopsy	-	iNO, O2	none	4 months: death
15	Flanagan, 2020 [13]	M	Birth	c.401 + 3 A > T	NA	AD	1	Cardiac catheterization	+/-	iNO, sildenafil, tadalafil, bosentan	none	5 years: moderate-severe PH
16	Hernandez-Gonzalez,2020 [1]	F	4 m	TBX4 deletion	NA	De novo	3	Echocardiography, CT, cardiac catheterization	+	endothelin receptor antagonist, sildenafil	none	5 years: resolution of PH
17	Hernandez-Gonzalez,2020 [1]	F	8d	TBX4 deletion	NA	De Novo	0	Echocardiography	+/-	sildenafil, systemic prostacyclin	none	4 months: clinical improvement
18	Karolak, 2020 [14]	F	Birth	Del 17q23.1q23.2	NA	De Novo	NA	Echocardiography, lung biopsy	NA		none	1 day: death
19	Bölükbasi, 2021 [15]	F	Birth	c.1112dup, SNP rs62069651-C	(p.Pro372Serfs*14)	AD	NA	Autopsy	NA		none	2 weeks: death
20	Bölükbasi ¸2021 [15]	M	Birth	c.1112dup, SNP rs62069651-C	(p.Pro372Serfs*14)	AD	NA	Autopsy	NA		none	2 weeks: death
21	Tsoi, 2022 [16]	F	Birth	c.576 C > A	p.(Tyr192* )	De Novo	NA	Echocardiography, cardiac catheterization, chest CT and lung biopsy	+/-	iNO, sildenafil,bosentan	none	7 years:restricted lung disease
22	Domingo, 2022 [17]	M	Birth	17q23 microdeletion	NA	NA	NA	Cardiac catheterization, chest CT	+/-	sildenafil, bosentan, intravenous treprostinil, milrinone, iNO, riociguat	none	21 months: moderate PH
23	Flanagan, 2023 [18]	M	Birth	c.401 + 3 A > T	chr17:59543302	AD	1	cardiac catheterization, chest CT	+/-	iNO, sildenafil, tadalafil, bosentan	none	3 years: chronic respiratory failure
24	Flanagan, 2023 [18]	F	Birth	c.401 + 3 A > T	chr17:59543302	AD	NA	Post mortem lung biopsy	NA		none	2 days: death

The genetic diagnosis was available in all patients, with 11 cases (46%) of *de novo* mutation.

Concerning the therapeutic approach to PH, data were available for 17/24 patients: 9 patients (37%) received iNO, 13 (54%) were treated with phosphodiesterase 5 inhibitor (sildenafil, tadalafil), 7 (29%) with endothelin receptor antagonist (bosentan), 4 (16%) with prostacyclin (epoprostenole, treprostinil), in 3 cases (12%) milrinone was added, 1 patient (4%) after failure of several drugs was treated with riociguat (an oral soluble guanylate cyclase stimulator ).Overall 3 (12%) patients needed ECMO. Ten patients (42%) died during neonatal period or first months of life despite maximal medical therapy with pulmonary vasodilatory drugs. Seven patients (29%) are on follow up for persistent moderate PH during childhood, 4 cases (16,5%) developed a chronic lung disease with chronic respiratory impairment and in 2 cases (8%) the PH resolved with clinical improvement.

## Discussion 

We report the case of a newborn presenting with refractory PH soon after birth, requiring advanced medical treatment with ECMO and a prolonged hospitalization. She was diagnosed with a clinical picture of PH associated with a novel variant of the *TBX4* gene (c.1160delC). We would want to emphasize the idea that in the event of a case of neonatal PH, one should consider TBX4.

*TBX4* has an early embryonic expression in pulmonary mesenchymal cells. Many experimental findings, drawn from cell and animal investigations, strongly suggest that *TBX4* is crucial for fetal development, including the formation of the parenchymal and vascular lungs and the formation of cartilaginous rings of the trachea and bronchi as well as the development of smooth muscle cells in the trachea [21, 22].

.

The ICPPS phenotype suggests that human *TBX4* mutations do not affect early steps in limb development, such as limb-bud initiation, but do have a profound effect at later stages. Anomalies of the posterior foot structures, such as short fourth and fifth rays, and patellar aplasia/hypoplasia are later signs of of TBX4 mutations, sometimes being manifest only in adolescence or adult life [5]. At present, there is no evidence for a genotype-phenotype correlation. Indeed intrafamilial variability of patellar, pelvic, and foot anomalies was found in SPS families investigated in the literature [5].

In recent years, *TBX4* has also been suggested as pathogenetic for pulmonary disease.

Several authors reported patients with idiopathic or familial pulmonary hypertension and identified heterozygous *TBX4* mutations. In some cases mutations were inherited from parents with ICPPS and without pulmonary hypertension [11, 23, 24].

Recently, *TBX4* mutations have been found in neonates with pulmonary hypoplasia and others developmental lung disease (acinar dysplasia, congenital alveolar dysplasia), supporting *TBX4*’s involvement in the embryonic development of the lung [9, 25].

From the review of the literature, it appears that the clinical picture associated with *TBX4* variants found in neonatal cases is severe, with PH requiring advanced medical therapy and a poor respiratory outcome, due to a severe lung disease, in those who survived.

Before starting therapy, a thorough history and physical examination, formal assessment of cardiac function, and diagnostic tests for the evaluation of PH pathogenesis/classification should be carried out at a center with experience [26].According to the guidelines of pediatric PH serial echocardiograms and cardiac catheterization are recommended for new diagnosis and t is advised to have another cardiac catheterization three to twelve months after starting treatment in order to assess response or in the event that clinical symptoms worsen [26].

In particular many patients present a biphasic clinical course, which includes severe hypoxic respiratory failure and PH at birth, that may respond to aggressive management, followed by persistence or relapse of PH in the first few months or years of life [2, 11]. In fact, *TBX4* is regarded as being in charge of the developmental lung problems connected to pulmonary hypertension, according to the most recent update on pediatric pulmonary hypertension [27].

In our patient, PH progressively improved and it was possible to switch from intensive ventilatory and continuous pharmacological treatment to spontaneous breathing and orally-given drugs, such as bosentan.

Our patient’s variant (c.1160delC) is novel and has never been described in the literature; it was inherited from the father with an autosomal dominance mechanism, causing the insurgence of a premature codon stop (p.Arg389fsTer27) with a consequent formation of a truncated protein.

This frameshift mutation adds a proximal stop codon, hence it is possible that the resulting protein has no function (null version). A recent study by Prapa et al. about genotype-phenotype correlation documented that haploinsufficiency is not the only cause of *TBX4* syndrome; increase of function is also a possibility. Indeed, the varied impact of pathogenic mutations found in important protein domains may help to explain some of the TBX4’s pleiotropic effects in lung illness [28]. 

To date, no certain data exist about the genotype-phenotype correlations of the lung manifestations.

Furthermore, the knowledge of clinical phenomenology of *TBX4* mutations in humans is expected to continue to increase, moving beyond bone problems to include vascular and parenchymal lung diseases.

The long-term outcome of infants with PH is primarily determined by their underlying diseases and the therapeutic interventions they receive at birth. In fact, the optimization of medical therapy since birth, including exposure to high oxygen concentrations, invasive mechanical ventilation, and ECMO, can play a fundamental role in short- and long-term outcomes [3, 29].

According to the recent guidelines on pediatric PH and as documented by our revision of the neonatal cases of PH, iNO is the most widely used drug in the treatment of neonatal PH [26]. Its efficacy is well documented although a rate of approximately 30% non-responders is reported [30]. In patients who do not respond to iNO, the best alternative is currently sildenafil [31]. Other current treatment options include milrinone, prostacyclins and bosentan [32–35].

## Conclusion

We described a complex case of severe neonatal persistent PH caused by a father-inherited novel mutation in the *TBX4* gene, in the absence of skeletal abnormalities. We faced with an unusual and complex case of “idiopathic” refractory PH, which needed the most support and a multidisciplinary team during the hospitalization and in the follow up.

Given the intricacy of the clinical course, the need for extensive genetic counselling, and the uncertainty surrounding long-term effects, the early detection of this condition was essential for the best care of our patient. We believe that it is crucial to include *TBX4* in extensive genetic studies of neonates with PH (combining a NGS panel and an array CGH), considering that *TBX4* mutations have been shown to play a variety of roles in diffuse and chronic pulmonary diseases, particularly in newborns. Being able to determine the best treatment options and establish the long-term prognosis is challenging and further research is required to determine the specific genotype-phenotype correlations.

## Data Availability

Not applicable.

## Abbreviations

ECMO: extracorporeal membrane oxygenation
ICPPS: ischio-coxo-podo-patellar syndrome
iNO: inhaled nitric oxide
NGS: next-generation sequencing
PH: pulmonary hypertension
SPS: small patella syndrome
*TBX4*: T-Box factor 4
